# Phylogeography and genetics of the globally invasive snail *Physa acuta* Draparnaud 1805, and its potential to serve as an intermediate host to larval digenetic trematodes

**DOI:** 10.1186/s12862-018-1208-z

**Published:** 2018-07-03

**Authors:** Erika T. Ebbs, Eric S. Loker, Sara V. Brant

**Affiliations:** Department of Biology, Museum of Southwestern Biology Parasite Division, Center for Evolutionary and Theoretical Immunology, University of New Mexico, 167 Castetter MSCO3 2020, Albuquerque, NM 87131 USA

**Keywords:** Invasion genetics, *Physa*, Physidae, Mitochondrial marker, Trematode, Parasite invasion, Parasite richness, Enemy-release

## Abstract

**Background:**

*Physa acuta* is a globally invasive freshwater snail native to North America. Prior studies have led to conflicting views of how *P. acuta* populations are connected and genetic diversity is partitioned globally. This study aims to characterize phylogeographic and population genetic structure within the native range of *P. acuta,* elucidate its invasion history and assess global patterns of genetic diversity. Further, using meta-analytic methods, we test the ‘Enemy-Release hypothesis’ within the *P. acuta* – digenetic trematode system. The ‘Enemy-Release hypothesis’ refers to the loss of native parasites following establishment of their host within an invasive range. Population genetic data is combined with surveys of trematode infections to map range-wide trematode species richness associated with *P. acuta,* and to identify relevant host-population parameters important in modeling host-parasite invasion.

**Results:**

Phylogenetic analyses using mtDNA uncovered two major clades (A & B). Clade A occurs globally while clade B was only recovered from the Western USA. All invasive populations sampled grouped within Clade A, where multiple independent source populations were identified from across North America. Significant population genetic structure was found within the native range of *P. acuta,* with some evidence for contemporary geographic barriers between western and eastern populations. Mito-nuclear discordance was found suggesting historical isolation with secondary contact between the two mitochondrial clades. Trematode species richness was found to differ significantly between native and invasive populations, in concordance with the ‘Enemy-Release hypothesis’. Further, our data suggests a positive relationship between nucleotide diversity of invasive populations and trematode prevalence and richness.

**Conclusions:**

This study includes a wider geographic sampling of *P. acuta* within its native range that provides insight into phylogeographic and population genetic structure, range-wide genetic diversity and estimation of the invasion history. Meta-analysis of *P. acuta –* trematode surveys globally is consistent with the ‘Enemy-Release hypothesis’. Additionally, results from this study suggest that host demographic parameters, namely genetic diversity as a proxy for population size, may play an essential role in how parasite communities assemble within invasive host populations. This knowledge can be used to begin to construct a framework to model host-parasite invasion dynamics over time.

**Electronic supplementary material:**

The online version of this article (10.1186/s12862-018-1208-z) contains supplementary material, which is available to authorized users.

## Background

Invasive species are an important consequence of global change [1], therefore understanding their origin and invasion history [2], as well as how genetic variation is partitioned across their range [3], is vital. Population genetics provides a powerful tool by which invasion processes can be revealed [2, 4]. Freshwater gastropods present an interesting case, as they are often dispersed passively and distributed patchily [5] in nature, but human activities (i.e. aquaria trade, boats) may greatly alter dispersal, population structure and consequently evolutionary dynamics [5, 6].

One such invader is *Physa acuta* [7], a globally invasive freshwater snail native to North America [8, 9], which now occurs on all continents except Antarctica [10, 11]. Remarkable reproductive plasticity has been reported within *P. acuta*, specifically in populations’ ability to alter the number of generations per year [12, 13], a characteristic that might contribute to the invasion success of *P. acuta*. *Physa acuta* is known to displace native gastropods to become the dominant species over very short periods of time [14–16]. For example, doubling times as short as 4 weeks have been recorded from European (invasive) *P. acuta* populations [17].

The invasion history of *P. acuta* is muddled by two centuries of taxonomic confusion (summarized by Wethington and Lydeard [10]), due in large part to plasticity in shell phenotype and overestimation of nominal diversity. Consequently, the timing of invasion and the connectivity of native and invasive populations remain unclear. Interestingly, *Physa acuta* was first described from its invasive range in France [7] over two centuries ago and it was not until 12 years later that it was described by Say [18] from Pennsylvania, USA, under a different name, *Physa heterostropha.* Nominal diversity of *P. acuta-*like snails reached at least eight species [19–21] prior to the inclusion of molecular genetic data [10, 22, 23]. Molecular genetic studies in addition to reproductive isolation experiments [8, 24] demonstrated the need to synonymize *P. heterostropha* (North America)*, P. virgata* (North America), *P. integra* (North America) and *P. cubensis* (Central and South America) to a single species, *Physa acuta* [7]*.* Taylor [21] placed *P. acuta* and closely allied species into the genus *Haitia*, based primarily on penial morphology, which has demonstrated phylogenetic utility [10, 21]. However, a molecular phylogeny [10] has shown that *Haitia* in the sense of Taylor [21] is not monophyletic. Molecular phylogenetic analyses have also supported that *P. acuta* occurs globally [10].

Efforts have been made to elucidate the population genetic structure of *P. acuta* at both regional [25–28] and global [11, 25] scales. These studies reconstructed the genetic structure of *P. acuta* primarily in its invasive range in Europe*,* with limited sampling from native populations. Characterizing population structure within the native range is foundational to resolving invasion history [29]. Limited sampling from native populations limit possible hypotheses relative to a likely invasion history scenario and identification of source populations. Further, those prior studies have presented conflicting results of how genetic varation is partitioned globally, and specifically whether genetic diversity is homogenous among native and invasive populations [11, 25, 28]. The primary objective of this study was to address current gaps in our knowledge of the genetic distribution of *P. acuta* by chacterizing range-wide population genetic and phylogeographic patterns, and reconstruct its invasion history.

Apart from its remarkable distribution, *P. acuta* offers a unique lens to understand host-parasite invasion dynamics, which are complex and largely overlooked [30–32]. Physid snails are important intermediate hosts to digenetic trematodes (Platyhelminthes, Digenea) [33, 34]. More broadly, snails act as first and second intermediate host to a large proportion of trematodes, over 10,000 species [35]. Despite the several papers discussing parasites in invasive species, surprisingly little is known about how host population dynamics might influence the invasion processes of their associated parasites [31, 32]. Following invasion, host species are expected to lose their associated trematode assemblages [36]. Parasite loss has been attributed to host invasive success, as per the ‘Enemy-Release hypothesis’ [37], which posits that release of regulation by natural enemies contributes to the rapid establishment of invasive populations. It is also expected that invasive hosts may acquire a new indigenous parasite assemblage within their invasive range. What shapes the assembly of the new parasite community is unclear [31]. Here we measure *P. acuta-*trematode species richness globally to test for release of digenetic trematodes within invasive populations and secondarily test for correlations among trematode infections and host population genetic parameters to identify factors that maybe relevant to host-parasite invasion, and discuss a framework to use these data to being to model invasion dynamics.

## Methods

### Specimen collection

**Fig. 1:**
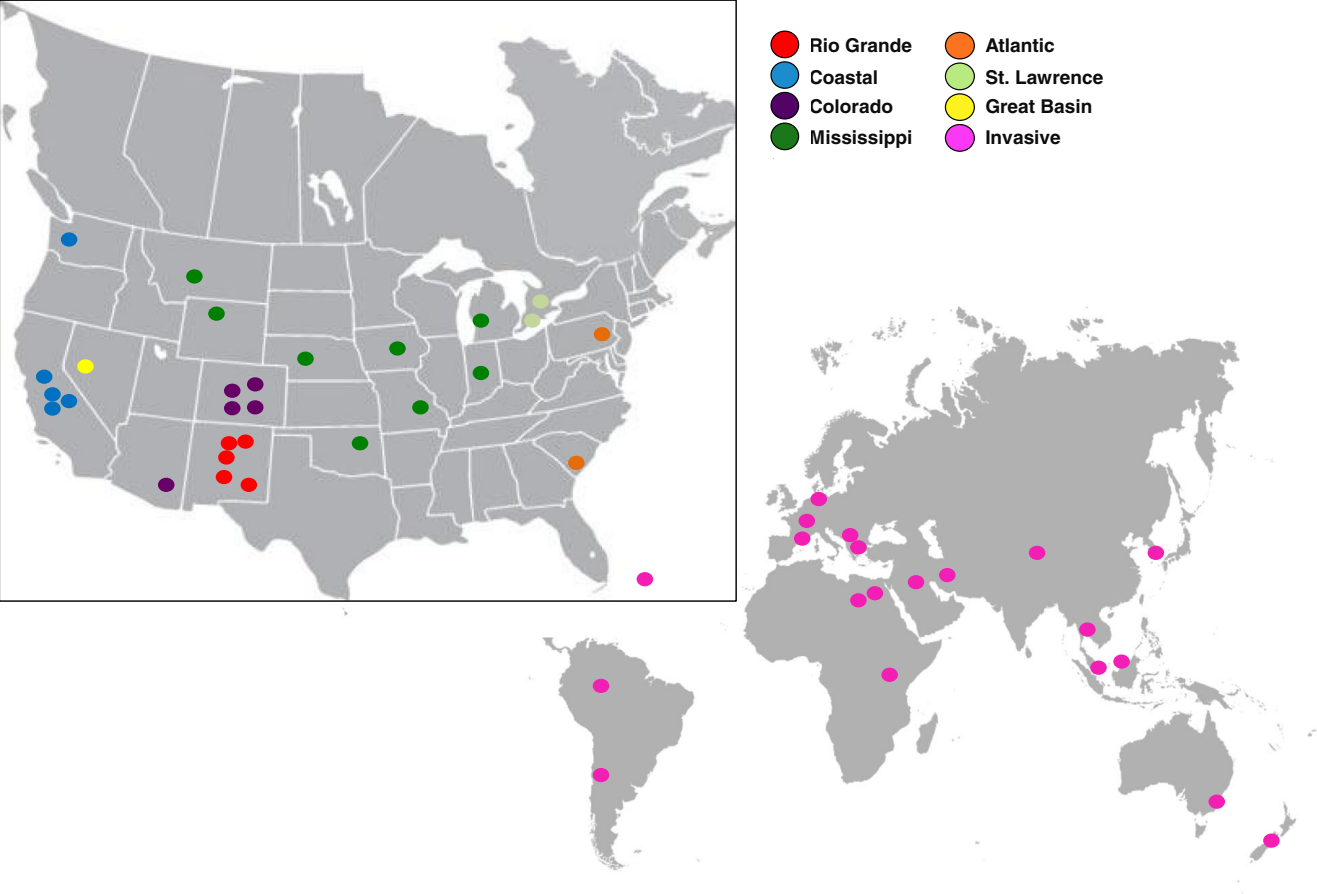
Map of sampled populations. Colors denote the Freshwater Ecological Complexes from which *P. acuta* populations were collected

*Physa acuta* populations were sampled opportunistically, between 1998 and 2015, to document the biodiversity of their trematode parasites. The specimens used in this study were collected from across North America (native range) as well as several invasive localities (locality details in Additional files 1 and 2) and represent *P. acuta* over a 17-year period (Fig. 1).

Snails were collected primarily from the waters edge using a mesh sieve or plucked from vegetation. The snails were first screened for trematode infection by leaving an individual snail overnight in the well of a 24-well cell culture plate filled with artificial spring water. Snails were considered infected upon visual confirmation of mobile trematode larvae (cercariae) using a dissection microscope. Cercariae and their snail hosts, as well as uninfected snails, were isolated and stored in 95% ethanol and deposited into the Museum of Southwestern Biology (MSB), Division of Parasites (Additional files 1 and 2).

To improve geographic sampling and ensure we were working with a monophyletic species, specimens from this study were combined in phylogenetic analyses with known *P. acuta* sequences published in the NCBI GenBank database (Additional file 1). The latter sequences were published between 2004 and 2016, from both native and invasive populations, and 159 physid sequences were downloaded and included in this study.

### DNA extraction and PCR amplification

A small piece of tissue was taken from the head foot of individual snails. DNA was extracted using the E.Z.N.A. Mollusc DNA kit (Omega Biotek) following the manufacturer’s protocol.

We sequenced two mitochondrial DNA loci (*cox*1 and *16S*) as they are the most abundant genetic markers available in the NCBI database and have been used successfully to recover population genetic variation within *P. acuta* [10, 22]. We also sequenced a nuclear locus (*ITS1*) to assess the potential for mito-nuclear discordance of the recovered subclades.

We sequenced 509 bases of the *cox*1 gene using the universal primers, LCO11490: 5’-GGTCAACAAATCATAAAGATATTGG-3′ and LCO2198: 5’-TAAACTTCAGGGTGACCAAAAAATCA-3′ [38] and 469 bp of *16S* rDNA using Brh: 5’–CCGGTCTGAACTCAGATCACGT-3′ and Arl: 5’–CGCCTGTTTAACAAAAACAT -3′ [39]. Additionally we sequenced the complete (495 bp) *ITS1* internal transcribed spacer region (ITS-1S: 5′- CCATGAACGAGGAATTCC CAG–3′, 5.8AS: 5′ –TTAGCAAACCGACCCTCAGAC – 3′) of a subset of the *P. acuta* individuals chosen to represent the recovered mitochondrial clades. Sequencing reactions were performed using the BigDye sequencing kit 3.1 (Applied Biosystems, Foster City, California, USA). Sequences were edited using Sequencher 5.3 (Gene Codes Corporation, Ann Arbor, MI, USA), and then aligned using ClustalW [40] and further aligned manually if necessary. GBlocks [41] was used to identify and remove difficult to align regions present within both the *ITS1* and *16S* alignments. Sequences generated during this study were submitted to GenBank under the accession numbers MF694400-MF694492, MG001330-MG001338 (Additional files 1 and 2). All alignments used in this study were deposited into TreeBase (www.treebase.org).

### Phylogenetic analyses

Phylogenetic analyses of *Physa* species were performed for the purposes of determining the in-group in all subsequent phylogeographic and population genetic analyses. *Aplexa elongata* was used as an out-group [10]. *Physa* phylogenetic datasets were analyzed separately as *cox*1 (509 bp), *16S* (611 bp) and *cox*1 + *16S* (1120 bp). Relatively few *ITS1* (525 bp) sequenes exist within the NCBI database (Additional files 1 and 2), and were therefore unavailable to be used in a concatenated analyses of *Physa* species. The T92 + G model of nucleotide substitution was used to model the *cox*1, *16S*, and *ITS1* datasets, and HKY + G was used to model the *cox*1 *+ 16S* dataset. Models were chosen based on the Akaike information criterion (AIC [42]) using JModelTest2 [43].

Phylogenetic analysis of *Physa* species did not recover a supported sister clade for *P. acuta,* therefore *Physa spelunca* was chosen as an out-group based on Wethington and Lydeard [10] and was used to root subsequent in-group analyses. Samples accessioned in NCBI that were identified as *P. acuta* via BLASTn search but had a genetic distance greater than 5% (distance between *P. acuta* and *P. spelunca*) were excluded from in-group analyses. In-group analyses included 151 individual snails and were performed to evaluate phylogeographic patterns within *P. acuta.* Each locus was treated as an independent dataset and analyzed separately. Optimal models of nucletotide substitiution were found to be the same per gene region as used for phylogenetic analysis of *Physa.*

Maximum Likelihood (ML), Minimum Evolution (ME) and Bayesian Inference (BI) were performed on each phylogenetic dataset. ME and ML analyses were performed in the program PAUP* 4.0 [44]. One thousand bootstrap replicates were performed within each gene tree analysis to statistically assess the resulting topologies.

Bayesian Inference (BI) was performed using the program MrBayes v. 3.2.6 [45], consisting of two replicated runs for each locus with four Markov chain Monte Carlo (MCMC) chains, one cold and three heated chains. Each analysis ran for 10,000,000 generations and was sampled every 1000 generations. The analysis was terminated when the standard deviation of the split frequencies was or fell below 0.01, supporting convergence. Likelihood parameters and convergence between runs were assessed using the program Tracer v.1.6 [46], based on EES values greater than 200. The first 2500 trees from each analysis were discarded as burnin. Resulting phylogenetic trees were visualized and manipulated using Fig Tree v. 1.3 [47] and MEGA 6 [48].

Recovered topologies were statistically compared to a null phylogenetic hypothesis (*H*_*O*_) using an approximately unbiased (AU) test [49], where per site likelihood scores were bootstrapped (10,000 replications) using the program CONSEL [50] to generate *p-*values. To test the ‘single source’ hypothesis we constrained all invasive individuals as monophyletic, with an Eastern USA origin (*H*_*O*_^1^)_,_ using the *cox*1 dataset. Secondly, to test for mito-nuclear discordance within *P. acuta,* we constrained the *ITS1* topology for complete concordance with recovered mitochondrial topologies (*H*_*O*_^2^). Constrained trees were created in Mesquite [51]. Per site likelihood scores were calculated in PAUP* [44] .

### Genetic diversity and population structure

To characterize range-wide population genetic diversity and structure we grouped native populations according to the freshwater ecological complex (FWEC [52]) from which they were collected. FWECs are based on delineations of freshwater ecoregions determined by fish distribution [53]. FWEC’s are a subset of ecoregions and are designated based on major habitat type and biological distinctiveness. We sampled 8 of the 10 North American FWECs [52]. We selected between 2 and 10 individual *P. acuta* from each population to be used for genetic analysis.

Genetic diversity summary statistics of *P. acuta* populations were estimated using DNAsp v. 5 [54] based on the *cox*1 and *16S* gene regions; number of unique haplotypes (*h*), number of segregating (polymorphic) sites (*S*), average number of nucleotide differences (K), haplotype diversity (*Hd*), nucleotide diversity (*π*) and Watterson’s estimator (Θ_w,_ per site) were calculated. The dataset of 151 individual *P. acuta cox*1 sequences consisted of 88 unique haplotypes. A minimum spanning network was constructed to view connections among haplotypes using the program PopART [55], http://popart.otago.ac.nz/). Relationships among haplotypes (185 bases) were also evaluated using BI, to determine ancestral relationships among haplotypes. Parameters for BI were identical to those used in analyses described previously.

To estimate overall range-wide genetic structuring of *P. acuta* (*cox*1) a one-way Analysis of Molecular Variance test (AMOVA [56]) was performed in Arlequin v. 3.5 [57]. Populations were partitioned by five geographical regions (North America, Eurasia, South America, Africa, and Australasia): Φ_CT_ estimates variation among the five regions sampled, Φ_SC_ estimates variation among localities within the regions and Φ_ST_ estimates variation from all samples across all localities sampled. A second AMOVA was preformed to assess population genetic structure within the native range of *P. acuta* partitioned by sampling localities and the eight FWECs sampled.

To further evaluate population genetic structure within *P. acuta,* pairwise Φ_ST_ [58] values were estimated from the *cox*1 dataset. Significance of Φ_ST_ values (*p* <  0.05) was determined by permutation tests of 10,000 random permutations, calculated in Arlequin v. 3.5 [57].

Pairwise uncorrected *p-*distances were calculated for *cox*1, *16S* and *ITS1* datasets within and between FWEC, countries within the invasive range, within and between the invasive and native ranges, and within and between recovered clades identified through MSN and phylogenetic analysis using MEGA 6 [48].

### Demographic analysis

We estimated changes in the effective population size (*Ne*) over time by fitting a Bayesian Skyline demographic model (BSP [59]) for each FWEC/geographic region, recovered clades and the total *cox*1 dataset in *BEAST v. 1.7.0 [60]. The HKY substitution model was used for two simultaneous MCMC runs for between 3000,000 and 80,000,000 iterations sampling between every 3000 and 10,000 steps. Analyses were run using three mutation rates of 1e-4 (0.04%), 1e-6 (1%) and 4e-6 (4%) per million year, chosen to reflect a range of possible rates of *cox*1 evolution based on other gastropod systems [61–64]. Convergence was checked (ESS of 200 or greater) and results visualized using Tracer v.1.6 [46]. BSP data from each analyzed dataset generated in Tracer v. 1.6 was exported and visualized in Microsoft Excel for comparison.

### *Invasion history and meta-analysis of the trematodes of P. acuta*

In conjunction with the molecular data generated by this study, a literature search was carried out to compile reports of *P. acuta* outside of North America.

ISI Web of Knowledge was searched for 1) all reports of *P. acuta* outside of North America and 2) surveys of trematode assemblages of *P. acuta* within both its native and invasive range. Data complied was published from 1805 up to the end of 2016, and recovered using combinations of keywords; *Physa acuta, Physa, Physella, Costella, Haitia,* trematode, parasites, invasive gastropod, malacological survey, trematode survey and cercarial survey. Further, studies cited by recovered articles were also included. In total the final dataset included 196 published reports of *Physa acuta* outside of North America and 7 trematode surveys from the native range of *P. acuta* and 15 within its invasive range. *Physa acuta* trematode surveys carried out by the authors for the purposes of this study were also added to the meta-dataset. From each trematode survey, observed species richness (*S*_*o*_) and prevalence (*pr*, percentage of patent infections) were calculated. *S*_*o*_ is defined as the number of different trematode species occurring within a surveyed host population [65]. Because of the difficulties associated with identifying larval trematodes a conservative approach to calculating *S*_*o*_ was taken: ‘species’ were considered unique based on trematode family. Consequently, reported measures of *S*_*o*_ from the native range are likely underestimates [34, 66]. Only patent infections, where trematode cercariae were being shed, were considered as infected. Evidence of metacercaria (trematode cysts) within *P. acuta* were not included as the relationships between trematodes and invertebrate second intermediate hosts are generally less specific [67, 68]. Genetic results from this study were used to assign host population parameters as regional estimates for each study used within the meta-analysis. For example, population genetic estimates from African populations were assigned to all *P. acuta* trematode surveys undertaken in Africa. Australasian samples were excluded from these analyses due to small sample size. Estimates of haplotype diversity (*Hd*) and nucleotide diversity (π), were assigned as population parameters. Time since invasion (*tsi*) was estimated by assigning each surveyed population into an ‘invasion phase’ category: phase 4: < 80 years since invasion, phase 3: 80–180 years since invasion, phase 2: 180–280 years since invasion and phase 1: 280 + (native range). ‘Invasion phases’ were determined based on published reports of *P. acuta* invasions and genetic evidence provided by this study. Pairwise correlation analyses of *S*_*o,*_ *tsi* and *pr* with *Hd,* π, and *Ɵ* were evaluated using Pearson’s correlation coefficient. *p*-values were adjusted for multiple comparisons using both Bonferroni and Benjamini-Hochberg corrections [69].

## Results

### Phylogenetic analyses

*Cox*1, *16S*, *16S* + *cox*1 and *ITS1* gene tree analyses (Additional files 3, 4, 5 and 6) of *Physa* species demonstrate that samples included in this study form a clade with *P. acuta* sequences published in the NCBI database (Additional files 1 and 2), and represent a single globally distributed species. All phylogenetic analyses failed to resolve sister relationships to *P. acuta*, similar to the findings of Wethington and Lydeard [10].

**Fig. 2:**
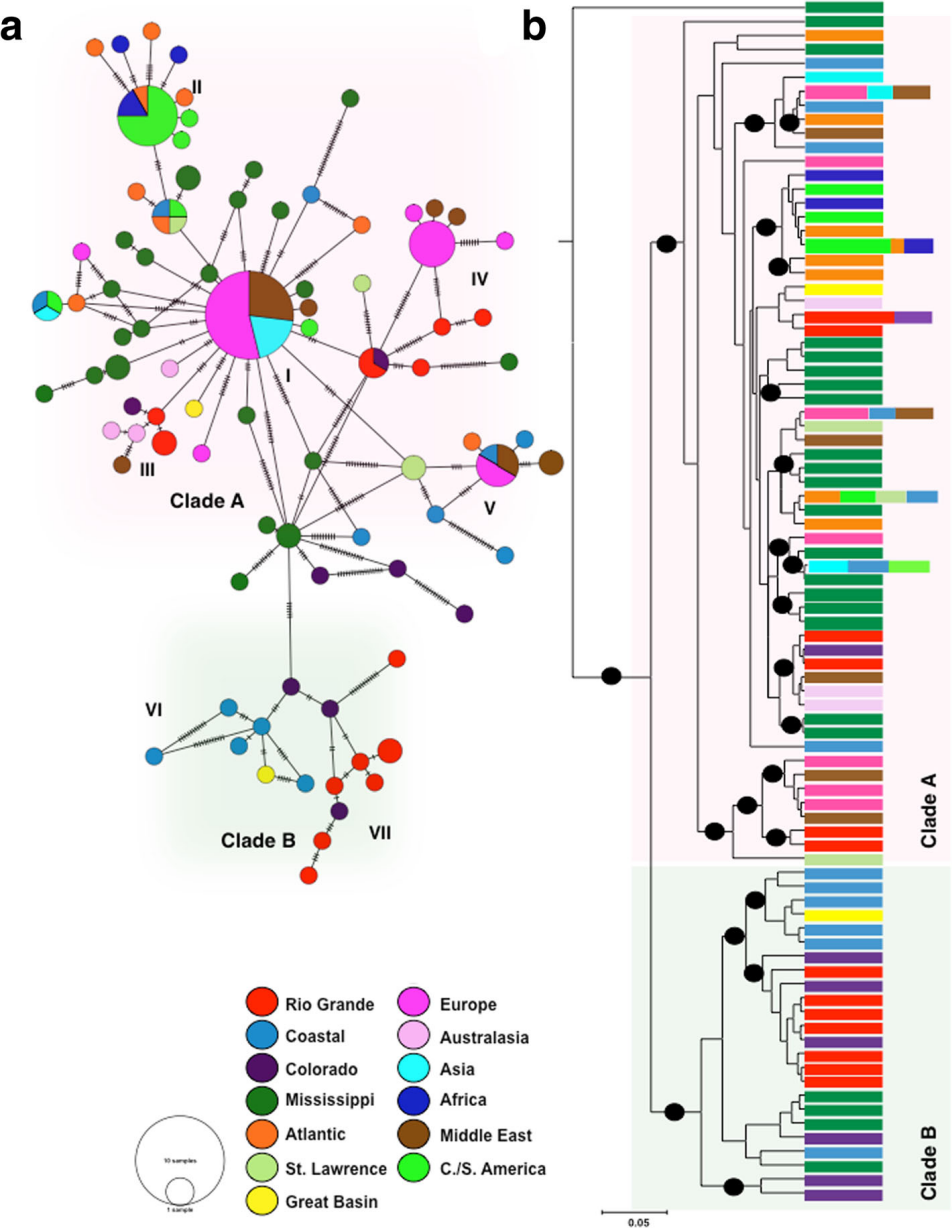
Relationships of recovered *cox*1 haplotypes. ***a.*** *minimum* spanning network of 88 *P. acuta* haplotypes. The network is shaded to depict the split between clade A (pink) and B (green), within each clade haplogroups are denoted as I –VII. **b.** BI phylogeny of all 88 haplotypes. Nodes with a posterior probability of ≥95% are denoted by a black circle. Haplotypes are colored by the FWEC from which they were collected

In-group analyses demonstrate phylogenetic structuring (Fig. 2, Additional file 6) and revealed two clades, referred to hereafter as clade A and clade B*.* Clades were moderately supported statistically for ME and ML analyses (*cox*1, 89/82; *16S,* 89/88) and posterior probabilities from BI were strong (*cox*1, 1; *16S*, 1). Clade A (*n* = 123) is the larger, containing all invasive populations sampled in addition to populations recovered across the native range. While some individuals from clade A and B were found sympatrically in the Western USA, clade B (*n* = 26) was only recovered west of the Rocky Mountains. Clades A and B were not recovered from the *ITS1* phylogeny, possibly suggesting mito-nuclear discordance (Additional file 5).

Phylogenetic analyses of the *P. acuta* in-group largely lacked resolution for terminal nodes across all genes samples. We therefore employed approximately unbiased topology tests to more robustly assess phylogenetic hypotheses. Using this method we rejected the null hypotheses of a single invasive origin (*H*_*O*_^1^,*p* = 2e-7) and of strict concordance of mitochondrial clades A and B within the *ITS1* dataset (*H*_*O*_^2^,*p* = 3e-5).

### Genetic diversity and population structure

Indices of genetic diversity (*cox*1 and *16S*) for each population sampled are summarized in Table 1. In comparing native to invasive range, greater genetic diversity (*Hd*_native_ = 0.97, *Hd*_invasive_ = 0.615; π_native_ = 0.0289, *π*_invasive_ = 0.0098) was found within the native range. Haplotype diversity is consistently high among FWECs sampled, often reaching 100% (Colorado, Coastal, Great Basin, Atlantic).

**Table 1:** Summary of range-wide population statistics, *cox1*

Region/Population	FWEC	N	H	Hd	S	K	π	Θw
Native Range								
USA								
Gila River, AZ	COL	3						
Denver Area, CO	COL	2						
Fremont Co., CO	COL	2						
Chaffee Co., CO	COL	1						
Gafield Co., CO	COL	1						
Mesa Co., CO	COL	1						
Rio Blanco Co., CO	COL	1						
Yuma Co., CO	COL	1						
*Total*		12	11	0.98	54	17.16	0.0337	0.035
Woodward Park, CA	COA	8						
Los Banos WMA, CA	COA	2						
Coyote Creek, CA	COA	3						
*Total*		13	13	1	69	18.45	0.0363	0.044
New Harmony, IN	MIS	2						
Okoboji Lake, IA	MIS	1						
Reynolds County, MO	MIS	1						
Medicine Lake, MN	MIS	1						
Bench Creek Pond, MT	MIS	10						
Mormon Lake, NE	MIS	12						
Big Horn River, WY	MIS	1						
Stillwater, OK	MIS	5						
*Total*		24	21	0.989	86	13.93	0.02736	0.0453
Stillwater NWR, NV	GRB	2						
*Total*		2	2	1	20	20	0.0393	0.0393
Carson Nat. Fr., NM	RIO	3						
Bosque Del Apache, NM	RIO	9						
Shady Lakes, NM	RIO	2						
Bitter Lake, NM	RIO							
*Total*		13	12	0.987	49	14.65	0.0278	0.029
Philadelphia, PA	ATL	2						
Charles Towne Landing SP, SC	ATL	7						
*Total*		9	9	1	33	10.58	0.021	0.024
Canada								
Point Peele NP, ON	STL	1						
Niagra River, ON	STL	3						
*Total*		4	3	0.833	14	8.167	0.016	0.015
*NR Total*		73	68	*0.973*	*45.29*	*14.74*	*0.0289*	*0.033*
Invasive Range								
Cuba	IR	3						
Chile	IR	10						
Lake Titicaca, Peru	IR	1						
*C&S AM Total*		14	6	0.604	13	2.879	0.0056	0.008
Kisumu, Kenya	IR	4						
*AF Total*		4	3	0.833	5	2.5	0.00493	0.005
France	IR	4						
Greece/Macadonia	IR	23						
Netherlands	IR	1						
*EU Total*		28	7	0.696	35	8.132	0.016	0.0177
New Zealand	IR	1						
Australasia	IR	2						
*NZ/OZ Total*		3	3	1	9	6	0.0118	0.012
South Korea	IR	1						
Thailand	IR	1						
Singapore	IR	3						
Maylasia	IR	3						
*AS Total*		7	2	0.286	6	1.714	0.0034	0.005
Iraq	IR	2						
Iran	IR	12						
*MD Total*		14	7	0.813	29	8.66	0.017	0.018
*IR Total*		70	28	*0.615*	*16.16*	*4.98*	*0.0098*	*0.011*
*Global Total*		143	96	*0.794*	*30.725*	*9.86*	*0.0193*	*0.023*

Regional abbreviations: *NR* native range, *IR* invasive range, *C&S AM* Central and South America, *AF* Africa, *EU* Europe, *NZ/OZ* New Zealand and Australia, *MD* Middle East, *FWEC* refers to Freshwater Ecological Complexes; *COL* Colorado *FWEC*, *COA* Coastal FWEC, *MISS* Mississippi FWEC, *GRB* Great Basin FWEC, *RIO* Rio Grande FWEC, *ATL* Atlantic FWEC and *STL* St. Lawrence FWEC. Population statistic abbreviations: *N* number of individuals sampled, H, number of haplotypes, *Hd*, haplotype diversity, S, segregating sites, K, average sequence divergence, π, nucleotide diversity, Θw, Wattersons estimator per site, Italicized numbers refer to population averages. (*) Indicates a statistically significant value (*P* <  0.05)

Within the *cox*1 and *16S* datasets, 88 and 43 unique haplotypes were recovered, respectively. More genetic variability was recovered from the *cox*1 dataset and haplotype relationships were estimated by constructing a Minimum Spanning Network (MSN; Fig. 2a). The MSN corroborates the split of clades A and B, however it also suggests that haplogroups may be further substructured. FWEC’s do not appear to be a primary factor in shaping the genetic structure of *P. acuta.* Clade A contains all of the sampled invasive haplotypes. Many of the haplogroups within clade A (I-V) are connected by haplotypes recovered from the Eastern United States (Mississippi and Atlantic FWECs), indicating that these regions are likely a source of invasions from the native range of *P. acuta*.

Clade B shows greater nucleotide diversity than clade A (even when invasive haplotypes are removed, π_CladeA_ = 0.019, π_CladeB_ = 0.024). All haplotypes were recovered from the Western United States (west of the Rocky Mountains). While sampling is not sufficient to know if Clade B is truly geographically restricted, our data suggest (1) clade B is a Western USA clade and (2) FWEC’s may play a greater role in shaping western populations of *P. acuta* (Fig. 2a, haplogroups VI and VII).

Relationships among haplotypes were also examined using BI analysis (Fig. 2b). Haplotype relationships are identical to those observed from the MSN, and the divergence of clade A and B is supported with 100% posterior probability. Invasive haplotypes group with multiple source populations, from across the native range. Together the MSN and BI analyses of haplotypes suggest that there have been several distinct invasion events to the Eastern Hemisphere from multiple sources across North America.

Overall population genetic structure of *cox*1 was evaluated by AMOVA (Table 2). Both range-wide and native-range AMOVA’s suggest a significant Φ_SC_ and Φ_ST_ (*p* <  0.001 and *p* <  0.001, respectively). In the native range 76.55% of genetic variation is attributed to within population variation, indicating that overall FWEC’s, and the biogeographic regions tested in general, are not important in structuring *P. acuta* populations.

**Table 2:** Analysis of Molecular Variance

Source of Variation	d.f.	% Variation	Fixation indices	*p* value
*Range Wide*				
Among Regions	6	3.19	ΦCT = 0.093	0.183
Among Populations within Regions	28	27.3	ΦSC = 0.282	< 0.001
Within Populations	110	69.52	ΦST = 0.305	< 0.001
*Native Range*				
Among Regions	6	4.27	ΦCT = 0.043	0.228
Among Populations within Regions	14	19.18	ΦSC = 0.20	< 0.001
Within Populations	55	76.55	ΦST = 0.235	< 0.001

Pairwise uncorrected *p-*distances within and between populations of *P. acuta* were calculated from the *cox*1 dataset (Table 3). Within the native range within population genetic distance averages 0.0292 (~ 3%), relative to 0.0102 (~ 1%) within the invasive range which are significantly lower (*p* = 0.0006). Within population genetic distances between the Western USA and Eastern USA (native range) vary significantly (*p* = 0.05), and average 0.0346 (~ 3.5%) and 0.022 (2.2%) respectively. Overall western populations exhibit the highest within population genetic distances while Africa and Asia demonstrate the lowest (x = 0.004, 0.4%).

**Table 3:** Pairwise genetic distances and Φ_ST_ values

	1	2	3	4	5	6	7	8	9	10	11	12	13
1. Rio Grande	*0.03*	0.0673	0.0167	**0.1571**	**0.2818**	**0.17422**	−0.0706	**0.2668**	**0.2396**	**0.2926**	**0.377**	**0.2613**	0.0288
2. Coastal	0.0366	*0.0386*	0.05412	**0.08**	**0.1557**	0.0269	**−0.2006**	**0.1841**	0.1509	**0.1573**	**0.2607**	**0.129**	−0.0085
3. Colorado	0.0338	0.0398	*0.0363*	**0.1694**	**0.3058**	**0.1857**	−0.0897	**0.3418**	0.24643	**0.35611**	**0.4045**	**0.3038**	0.0623
4. Mississippi	0.34	0.0364	0.0387	*0.0288*	**0.0969**	0.01138	0.022	**0.0983**	0.0745	0.00036	**0.245**	**0.0662**	−0.0125
5. Atlantic	0.0356	0.0364	0.0418	0.0283	*0.0214*	0.0773	0.2032	**0.1869**	**0.17405**	**0.1216**	0.0944	**0.1189**	0.0199
6. St. Lawrence	0.0295	0.0305	0.0348	0.0241	0.0211	*0.0166*	0.1199	0.0586	0.2199	**0.2518**	**0.4667**	−0.006	−0.0158
7. Great Basin	0.031	0.0328	0.0346	0.0327	0.0337	0.0281	*0.0333*	0.2469	0.2587	0.4986	0.4948	0.2333	−0.1357
8. Europe	0.0298	0.0325	0.0366	0.0251	0.023	0.0175	0.0288	*0.0165*	0.1726	0.0994	**0.3923**	0.0291	0.0932
9. Australasia	0.031	0.035	0.0373	0.025	0.022	0.0186	0.0311	0.0184	*0.012*	0.4158	**0.6129**	0.1178	−0.0355
10. Asia	0.0249	0.027	0.0318	0.017	0.0143	0.0116	0.0233	0.012	0.01	*0.003*	**0.7128**	0.0567	**0.13**
11. Africa	0.0343	0.036	0.0414	0.0277	0.016	0.02	0.0327	0.0221	0.02	0.01375	*0.005*	**0.383**	0.2029
12. Middle East	0.0317	0.0332	0.0378	0.0258	0.0228	0.0178	0.031	0.0181	0.019	0.0124	0.0228	*0.0187*	0.06245
13. S. & C. America	0.0311	0.0323	0.038	0.0243	0.0146	0.0173	0.0286	0.019	0.0174	0.0103	0.0056	0.0195	*0.0058*

Pairwise within and between FWEC/region genetic distances are shown in the lower diagonal. Within genetic distances are italicized. Pairwise Φ_ST_ values are shown in the upper diagonal, values with a *p*-value ≤0.05 are bolded

Pairwise genetic distances and Φ_ST_ values were calculated (Table 3) and assessed statistically (only significance values for Φ_ST_ are shown). The Mississippi FWEC, the largest complex sampled, shows significant (*p* <  0.05) genetic differentiation from four of the six other FWECs (Rio Grande, Coastal, Colorado and Atlantic) as well from Africa and Middle East. Similarly, the Atlantic FWEC shows significant (*p* <  0.05) genetic differentiation from four of the six other FWECs (Rio Grande, Coastal, Colorado and Mississippi) as well from Europe, Australasia, Asia and the Middle East.

### Demographic and invasion history analyses

**Fig. 3:**
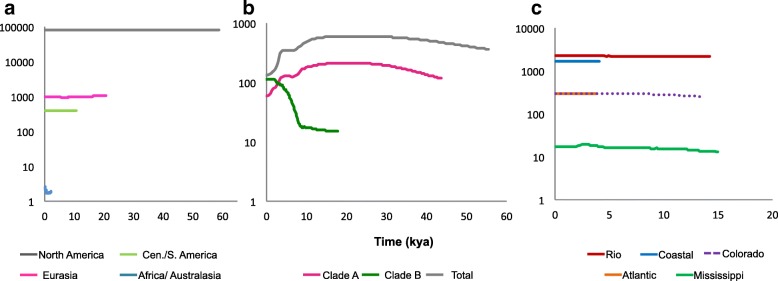
Bayesian Skyline Plots. Effective size over time plots estimated in BEAST using a Bayesian Skyline prior using a mutation rate of 1% (1e-6) per million years. Mean values are shown and confidence values were excluded for clarity. The x-axis indicates time in ‘thousand years ago’ (kya). **a.** Range-wide estimates for North America (grey, *n* = 79), Central and South America (lime green, *n* = 13), Eurasia (pink, *n* = 49) and Africa and Oceania (blue, n = 7). North America dataset ran for 50,000,000 generations, Eurasia 20,000,000 generations, Central/South America and Africa/Australasia ran for 5,000,000 generations. **b**. Estimates of recovered Clades, Clade A (pink, *n* = 116) and B (dark green, *n* = 33) compared to total (grey, *n* = 149). The total dataset and Clade A ran for 80,000,000 generations, Clade B ran for 10,000,000 generations. **c.** Estimates by FWECs sampled: Rio Grande (red, *n* = 16), Coastal (blue, n = 13), Colorado (purple, n = 11), Atlantic (orange, *n* = 9), Mississippi (green, *n* = 26), the Great Basin and St. Lawrence FWECs were excluded because of low sample size. All FWEC datasets were run for 5,000,000 generations, with the exception of Atlantic (3000,000). Bayesian skyline plots were constructed in TRACER, the data was exported and visualized using Excel

Bayesian Skyline Plots (Fig. 3, Additional file 7) estimated changes in effective population sizes over coalescent time. Results based on a 1% change per million year rate of *cox*1 evolution (Fig. 3) largely matched estimates using 0.04 and 4% change per million year (Additional file 7). Regionally (Fig. 3a) *Ne* is greatest within North America and has been stable over the past 60,000 years. Populations from the invasive range suggest much smaller effective sizes, congruent with previous analyses of genetic diversity. Eurasian and African/Australasian populations indicate a possible recent population expansion. When Clades A and B are compared (Fig. 3b), Clade A and the total *P. acuta cox*1 dataset suggests a recent population decline which is likely an artifact of population subdivision. Heller et al. [70] showed subdivision can confound coalescent estimators of *Ne* and result in an erroneous signal of population decline. There is no evidence (Fig. 3c) of population decline within the native range. There is evidence to suggest that Clade B has recently undergone a demographic expansion (within the past 10,000 years), both clades A and B have comparatively similar contemporary *Ne.*

### *Invasion timeline and meta-analysis of the trematodes of P. acuta*

**Fig. 4:**
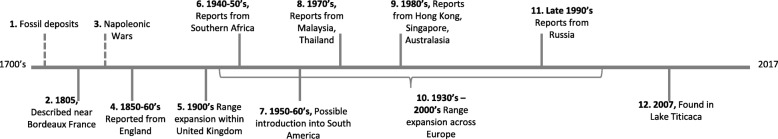
Invasion timeline of *Physa acuta.* 1. Absence of *Physa acuta* shells from European fossil deposits older than 18^th^ Century [115]. 2. Described by Draparnaud [7] near Bordeaux, France. Anderson [87] posits likelihood of introduction from Mississippi USA via the cotton trade. 3. Trade between USA and France ends during Napoleonic wars, trade with England and USA begins. 4. First reports of *P. acuta* in England [88, 89]. 5. Reports of *P. acuta* all over United Kingdom by the later half of 20^th^ Century [116–119] 6–12. Primary citations for these reports are listed in Additional File 7

Based on reports from the literature an invasion timeline is summarized in Fig. 4 (full bibliographic information can be found in Additional file 8). Findings from this literature search informed subsequent time since invasion analysis.

*Physa acuta* trematode surveys from both the native (*n* = 7) and invasive (*n* = 15) range were complied to estimate range-wide species richness (Table 4). Average *S*_*o*_ within the native range (*S*_*o*_ = 3.25) was significantly greater (*p* = 0.0016) than within the invasive range (*S*_*o*_ = 0.3125). Western European (*n* = 2) and Iranian (n = 1) populations of *P. acuta* were the only invasive populations surveyed where patent infections were reported. Prevalence varied significantly (*p* = 0.04) among invasive (0.00319%) and native ranges (0.2%). Variables were analyzed using Pearson’s correlation coefficient (ρ), summarized in Table 5. All comparisons between *So, tsi, pr* and the demographic variables (*Hd, π, Ɵ*) resulted in significant unadjusted *p*-values, with the exception of *pr* and *Hd*. To correct for multiple comparisons, *p*-values were adjusted using BH and Bonferroni methods (Table 5). Following adjustment, *So, tsi* and *pr* were found to significantly correlate with *π* and *Ɵ* (p_BH_ = 0.003, p_BH_ = 0.000, p_BH_ = 0.033, respectively). *π* and *Ɵ* were found to have a correlation coefficient of 1 (Table 5). There is a strong positive relationship between *tsi, π* (ρ = 0.97) and *So* (ρ = 0.8) and a moderate correlation with *pr* (ρ = 0.57), supporting the prediction that genetic diversity increases with time since invasion. In total, these results suggest a significant relationship between background genetic variation, a proxy for population size, and the richness of trematode assemblages.

**Table 4:** Reports of infection and trematode species richness of *Physa acuta* as first intermediate

Country	Citation	# Individuals Surveyed	# Patent Infections	Prevalence	*So*
*Native Range*					
New Mexico (Bosque del Apache)	This study	1200	78	0.065	5
New Mexico (Eagles Nest)	This study	65	5	0.07	2
Nebraska	This study	120	16	0.13	4
Montana	This study	165	3	0.018	2
Wyoming	This study	26	8	0.31	1
Colorado	This study	300	5	0.017	3
Mexico	Barragán-Sáenz et al. 2009	496	109	0.22	5
*Invasive Range*					
New Zeland	Mitchell & Leung 2015	810	0	0	0
Benin	Ibikounlé et al. 2009	345	0	0	0
Czech Republic	Faltýnková 2005	57	0	0	0
Egypt	Abo-Madyan et al. 2005	na	0	0	0
Egypt	Bin Dajem 2009	704	0	0	0
Egypt	Dahesh & Farid	na	0	0	0
France	Gerard et al. 2003	413	1	0.0024	1
Germany	Faltýnková and Hass 2006	141	0	0	0
Iceland	Skinnerson et al. 2008	737	0	0	0
Iran	Athari et al. 2006	3560	8	0.0022	1
Kenya	Loker & Laidemitt, unpublished data	589	0	0	0
Morocco	Laamrani et al. 2005	na	0	0	0
New Zeland	This study	1480	0	0	0
Spain	Toledo et al. 1998	2717	1	0.00037	1
Zambia	Phiri et al. 2007	9	0	0	0

*na* data not given, *S*_*O*_ observed species richness calculated by the number of unique trematode families observed

**Table 5:** Matrix of correlation coefficients.

	*S*_*O*_	*tsi*	*pr*	*Hd*	π	Ɵ
*S*_*O*_		0.8	0.77	0.54	0.76	0.77
*tsi*	**0.0025**		0.57	0.46	0.97	0.98
*pr*	**0.0039**	0.032*		0.44	0.55	0.56
*Hd*	0.0614	0.06	0.096		0.5	0.5
π	**0.003**	**0**	0.033*	0.061		1
Ɵ	**0.003**	**0**	0.033*	0.061	**0**	

Pearson’s correlation coefficients are shown in the upper diagonal and corresponding *p-*values, adjusted using Benjamini-Hochberg (BH) correction, are shown in the lower diagonal. *p-*values ≤0.05 are bolded. *Indicates *p-*values that significant under the BH correction, but not significant under the more conservative Bonferroni correction

## Discussion

### Range wide population genetic patterns

Our results propose different population genetic patterns between the native and invasive range of *P. acuta*. While haplotype diversity within the invasive range is not low relative to other invasive snails, like *Melanoides tuberculata* [6] or *Haminoea japonica* [71] within their invasive ranges, by comparison there is an approximately 20% reduction in *Hd* relative to the native range. Our analyses reveal that invasive populations have substantially less mtDNA diversity relative to native populations (Table 1), a finding supported by numerous studies of invasive gastropods [6, 71]. Interestingly however, this result is in contrast to recently published work on *P. acuta*. Bousset et al. [11] used 9 microsatellite loci to estimate range-wide patterns of genetic diversity and reported no difference in neutral genetic variation between the native and invasive ranges. These contradictory results are likely explained by the fact that the Bousset et al. [11] study was limited to five native populations, four of which were from the Eastern USA where many invasive haplotypes likely originated. Consequently, Bousset et al. [11] did not recover similar amounts of genetic diversity revealed by this study. Discordance of mtDNA and microsatellite data is not uncommon [72] and both reveal aspects of invasion history. Mitochondrial markers can often better detect subtle genetic subdivision, due to reduced effective population size and lack of recombination [72–74]. Consequently, a genealogical approach utilizing mtDNA markers is frequently used to characterize population genetic structure and identify source populations [6, 71, 75]. Our findings based largely on mtDNA, combined with those of Bousset et al. [11], suggest *P. acuta* is overall a genetically diverse species, relative to other invasive gastropods [6, 76, 77]. However, this study reports significant genetic structuring within the native range and between some native and invasive populations that has not been previously reported by other investigators. Further, previously unknown mito-nuclear discordance (Fig. 2, Additional files 5 and 6) suggests historical isolation followed by secondary contact has likely occurred among native Western and Eastern North American populations.

### Genetic structure within the native range and biogeographic considerations

Previous studies have hypothesized an Eastern USA origin for *P. acuta* [8, 11]*.* Our data suggest that sampled genetic diversity is comparable (marginally greater in Western USA) across the native range with evidence of geographic structuring between the Eastern and Western USA populations. Phylogenetic analyses suggest the existence of two clades (A & B) within the native range, interestingly these clades are sympatric in the Western USA, while clade B appears to be restricted to Western USA. Field surveys and subsequent molecular analysis reveal that individuals belonging to clade A and/or B can be collected side by side from the same water body in New Mexico, Colorado, California and Nevada. Phylogenetic analysis (Additional files 3, 4, 5 and 6) did not result in sufficient resolution to reveal the geographic origin of *P. acuta,* however increased sampling of western populations fails to support the Eastern origin hypothesis [8, 9, 11]. Several individuals (S94 & S22) collected from California (Coastal FWEC) and one (S17) from New Mexico (Rio Grande FWEC) were close matches genetically to *P. acuta,* but for *cox*1 had greater than 5% *p-*distance, our designated in-group cut-off. Additionally the presumed sister species *P. spelunca* has only been recovered from Wyoming [78] and did not have support as the sister species to *P. acuta* from our analyses. Anecdotally, recent molecular genetic studies of *Physa* [79–81] may demonstrate a greater number of independent taxonomic units from Western waterways relative to the Eastern USA, suggesting that biogeographically the west may have been important in physid diversification*.*

This study is the first to compare and assess discordance among nuclear and mitochondrial markers within *P. acuta.* Discordance between *ITS1* and mitochondrial phylogenies could suggest incomplete lineage sorting, though more likely is a recent history of genetic exchange between once isolated populations. Clades A & B were not recovered from the *ITS1* gene tree (Additional file 5), and when the largely unresolved *ITS1* phylogeny was constrained to match the mitochondrial topology a significantly (*p* = 3e-5) worse phylogeny was recovered. Gene trees of the non-recombining mitochondrial markers may suggest historical isolation between the Western and Eastern USA. It may be hypothesized that the Rocky Mountains, or associated waterways, have played a role in restricting gene-flow, which has been shown relevant in structuring populations of other freshwater organisms [82–84]. It can also be hypothesized that anthropogenic dispersal within North America or subsequent re-introduction from the invasive range into the Western USA, has led to reticulation of the two clades [85], and contemporary admixture within the Western USA. Another possible line of evidence supporting this hypothesis is the recent discovery of two distinct mitochondrial genomes, varying in size (Isolate A 14,490 vs Isolate B, 14,314 bp) and 37 indels, collected from the same pond in New Mexico [86]. *cox*1*, 16S* and *ITS1* sequence from Nolan et al. [86] was included in our phylogenetic dataset and consistently parse out with clade A (Isolate A) and B (Isolate B). At this time there has been no investigation into the biological relevance or functional consequences of Isolate A versus B. Mitochondrial genome divergence may in fact be a relic of once isolated populations conferring no functional differences. The fact that invasive haplotypes have only been recovered from clade A is an intriguing pattern warranting further investigations.

### *Invasion history of Physa acuta*

Figure 4 summarizes records of *P. acuta* globally (see Additional file 8 for full bibliographic information). These records, combined with population genetic inference, lead to a hypothesized route of invasion. The first record of *P. acuta* outside of North America was its initial description in France in 1805 [7], over 200 years ago. It is has been hypothesized by others, and supported genetically here, that these were the first invasive populations of *P. acuta*. Anderson [87] was the first to posit that the *P. acuta* discovered by Draparnaud [7], in the Bordeaux region of France, were introduced via the cotton trade from Mississippi USA during the 18^th^ century. Trade between France and the USA ceased during the Napoleonic wars and began between USA and England, where records of *P. acuta* appear in the mid 1800’s [88, 89]. Western Europe (England and France) likely maintained the first invasive populations of *P. acuta.* However, less than 100 years later, populations were reported from Eastern Europe, Africa, Asia, South America, the Middle East, the Caribbean, New Zealand and Australia. Prior studies have suggested that the subsequent global invasion resulted from the spread of early Western European colonizers [87, 90]. If this was the case we would expect 1) clustering of all invasive populations with Western European haplotypes and 2) a stepping stone like model of haplotype relationships [91, 92]. This model was not supported by any of the performed population genetic analyses and further, an approximately unbiased (AU) [49] topology test specifically rejected the monophyly of invasive populations (*p =* 2e-7).

Our data supports that there have been multiple independent invasions into Western Europe, likely from different sources (Fig. 2). Data from this study suggest that some European populations are closely allied with populations in Asia and the Middle East and share the most common invasive haplotype. We infer that this shared haplotype [93] is related to the initial invasive populations of *P. acuta* in Western Europe roughly 200 years ago, and has subsequently spread east. It is more parsimonious to hypothesize that genetically depauperate European populations expanded east than to assume that identical haplotypes from a genetically diverse source (North America) founded three distinct invasive regions.

Samples from Africa, South America and the Caribbean are related and are likely the result of an independent invasion event, though it is currently not possible to determine the directionality without more samples. *Physa acuta* invasions into Africa have been more recent relative to European invasions. Extensive malacological surveys across the African continent suggest the African invasion occurred in the range of 1940–50’s, with populations reported from South Africa [15, 94, 95], Namibia [96], Morocco [97], Nigera [98], and Kenya [99]. Records suggest a similar time frame for the South American and Caribbean invasion [100, 101]. Surveys by Albrecht et al. [102] suggest that Lake Titicaca (Peru & Bolivia) was colonized 20 years ago.

Our data suggests haplotype connections between invasive populations and Clade A individuals collected in the Western and Eastern USA and Canada. While our data supports the initial invasions from the Southeastern USA to Western Europe, we also find evidence of haplotype connections between the Coastal FWEC (California) and the Middle East and Eastern Europe. Currently, based on our sampling it is unclear if there are in fact Western source populations, or if this relationship is the result of the re-introduction of invasive *P. acuta* from other continents into the Western USA. Our data supports that all source populations have originated from Clade A, however, it remains unclear if Western haplotypes of Clade A originated from the Eastern USA or are the result of secondary contact from invasive populations, though these scenarios are not mutually exclusive. Increased sampling within the western range is necessary to fully elucidate the source of Clade A members recovered from the Western USA. Founding events, within and from the native range, are likely the result of the aquaria or aquatic plant trade as posited by multiple investigators [11, 15, 87, 103].

### Host-parasite invasion dynamics

Combining published reports of *P. acuta* occurrence with our genetic data we found a significant relationship between genetic diversity (*π* and Ɵ) and trematode prevalence (*pr*), and richness (*S*_*O*_). Nucleotide diversity (*π*) is a better measure of background genetic variation than haplotype diversity, as it is more robust to the stochastic effects of drift [104]. Higher values of haplotype diversity relative to nucleotide diversity were recovered from the invasive range suggesting rapid expansion from founding populations [104].

We also found that time since invasion (*tsi*) significantly correlated with *π,* Ɵ as well as *pr* and *S*_*O.*_ Of all the invasive populations screened for trematodes and included in this study there were only three reports of *P. acuta* shedding larval trematodes (cercariae) within its invasive range (Table 4); France [105], Spain [106], Iran [107]. None of the cercariae recovered from invasive *P. acuta* populations were characterized genetically, as such only broad taxonomic distinctions can be made; Echinostomatidae (France [105]), Strigeidae (Iran [107]) and Xiphidiocercous cercariae (Spain [106]). Determining origin of infection of invasive *P. acuta* (i.e. spillback versus spillover) is inhibited by a lack of taxonomic resolution. Inclusion of parasite genetic data is required to better understand *Physa-*trematode invasion dynamics.

Populations from Western Europe (presumably the first invasive populations) and allied populations from the Middle East have the greatest *π* of invasive populations sampled. These data suggest that richness of trematode infection increases positively with background genetic variation and time since invasion, and leads to the prediction that over time as invasive *P. acuta* populations increase in size their role in local trematode transmission will increase. Meta-analysis was limited by population genetic sampling within the invasive range, which will be necessary to more robustly model the relationship between genetic diversity and trematode prevalence.

Overall results from meta-analyses support the ‘Enemy-Release’ hypothesis [36, 37]. There are numerous explanations for this within the *P. acuta –* trematode system. Firstly, most founding populations likely consisted of eggs or young snails, as common with the aquarium trade, and were therefore uninfected. Secondly, there might be incompatibility among native parasites and invasive host populations. An additional ecological explanation is that invasive *P. acuta* populations often establish in highly modified habitats (i.e. drainage ditches, man-made water-bodies [14, 87]), which may not be suitable to sustain a diversity of trematode life cycles. As invasive populations spread over time into a broader range of habitat types, one would expect interaction with wildlife trematode fauna to become more frequent with a greater potential for native parasites to establish in *P. acuta*. For example, in countries such as England and Germany, where the invasion history of *P. acuta* is well documented, initial reports of *P. acuta* are from habitats such as botanical gardens that are known to import aquatic vegetation, but less than a decade later, *P. acuta* is reported from natural habitats (summarized by Vinarski [108]).

Apart from directly hosting trematodes, invasive *P. acuta* populations might play a role in diluting or enhancing transmission of indigenous parasites in several ways. *Physa acuta* may act as a decoy host [109–111] to indigenous trematode species, as it is an efficient competitor to native snails often displacing them to become the dominant gastropod within the community [14, 16]. Dobson [16] showed that following its establishment in Mozambique, *P. acuta* overtook *Bulinus forskalii,* a primary transmitter of human schistosomiasis (*Schistosoma haematobium*), as the dominant snail*.* It has been shown experimentally *Schistosoma haematobium* can penetrate *P. acuta* in Nigeria, though the parasite cannot reach patency [112]. There are also numerous reports of invasive *P. acuta* acting as a reservoir for larval parasites, specifically as a second intermediate host to trematodes. Similarly, non-host specific larvae of the invasive nematode *Angiostrongylus* (=*Parastrongylus*) *cantonensis* were reported in Saudi Arabia [113] and Egypt [114] from *P. acuta.*

Host-parasite invasion dynamics are often underappreciated when considering the impact of invasive species, especially when the associated parasites are not causing notable pathology. Most commonly it seems hosts acquire a distinct assemblage of indigenous parasites within their invasive range [36] over time, and the consequences to local transmission and biodiversity are not clear. While the primary goal of this study was to characterize the population genetic structure and invasion history of *P. acuta,* we aim to begin to use this system to address the role of host demographics in the acquisition of local parasite assemblages specifically, to identify potential predictors of parasite invasion success for the purposes of modeling host-parasite invasion over time.

## Conclusions

This study provides improved sampling of *Physa acuta* within its native range, where mtDNA analyses reveal two phylogenetic clades (A & B) and previously unknown population structure between the Eastern and Western USA. Increased sampling of native populations allowed the estimation of the invasion history of *P. acuta.* We report evidence for the occurrence of multiple independent invasion events from different source populations across the USA and Eastern Canada. The initial invasion into Western Europe occurred over 200 years ago, with more recent invasions into the Southern Hemisphere (South America, Africa) occurring within the last 20–80 years.

We also examined the relationship between *tsi, pr, S*_*o*_ and host-population genetic parameters (*Hd, π, Ɵ*) to identify parameters of relevance for modeling host-parasite invasion dynamics over time. We found a significant positive relationship among *π* with trematode *pr* and *S*_*o*_. These data suggest that, (1) host demographic parameters might be integral in the assembly of parasite communities within invasive host populations and (2) background genetic diversity may be an important parameter to consider in future efforts to model host-parasite invasion dynamics. Overall, our data supports predictions based on the ‘Enemy Release hypothesis’ [36, 37]. Hosts, particularity snail intermediate hosts, are substantially easier to sample and monitor over time and space relative to their associated trematode assemblages and may prove a valuable and tractable tool to model parasite invasion.

## Additional files

Additional file 1: *Physa acuta* locality and database information. (XLSX 27 kb)

Additional file 2: Non-*Physa acuta* locality and database information (XLSX 13 kb)

Additional file 3: CO1 phylogeny of *Physa* estimated using Bayesian inference. Posterior probability values ≥.95 are shown. Taxon names are reflective of the names assigned to sequences in the original NCBI records. (PDF 14 kb)

Additional file 4: Concatenated 16 + *cox*1 phylogeny. Branch support values ≥.95/70 (PP/BS) are shown. Taxon names are reflective of the names assigned to sequences in the original NCBI records. (PDF 165 kb)

Additional file 5: *ITS1* phylogeny estimated using Bayesian inference. *Physa acuta* samples are colored according to the clade they were recovered from based on mitochondrial gene tree analysis: pink = Clade A, green = Clade B. *Physa acuta* taxa labeled as black did not group within *P. acuta* based on mitochondrial gene trees, and were excluded from all in-group analyses (S17, S120 and all *P. zionis* samples). *ITS1* data only exists for HQ283259 and Pacuta_18S/ITS and were therefore not included in mitochondrial gene tree analyses. Posterior probabilities are illustrated as branch support values. (PDF 143 kb)

Additional file 6: 16S in-group analysis. Taxa are colored according to the FWEC they were collected from. Phylogeny (A) was generated using ML methods and phylogeny (B) was generated using BI. Branch support values ≥70 bootstrap (A) and ≥ .95 posterior probabilities (B) are denoted by a black circle. (PDF 188 kb)

Additional File 7: Bayesian Skyline Plots. Effective size over time plots estimated in BEAST using a Bayesian Skyline prior, mean values are shown and confidence values were excluded for clarity. Three mutation rates (per million year): 1% (solid line), 0.04% (dashed line) and 4%(circle line) were tested. (A) Range-wide estimates for North America (NAM, dark grey, *n* = 79), Central and South America (SAM, lime green, *n* = 13), Eurasia (EUR, pink, *n* = 49) and Africa and Oceania (AFOC, blue, *n* = 7). North America dataset ran for 50,000,000 generations, Eurasia 20,000,000 generations, Central/South America and Africa/Oceania ran for 5,000,000 generations. B. Estimates by FWECs sampled: Rio Grande (RIO, red, *n* = 16), Coastal (COA, blue, *n* = 13), Colorado (COL, purple, *n* = 11), Atlantic (ATL, orange, *n* = 9), Mississippi (MISS, green, *n* = 26), the Great Basin and St. Lawrence FWECs were excluded because of low sample size. All FWEC datasets were run for 5,000,000 generations, with the exception of Atlantic (3000,000). C. Estimates of recovered Clades, Clade A (A, aqua, *n* = 116) and B (B, dark green, *n* = 33) compared to total (TOT, light grey, *n* = 149). The total dataset and Clade A ran for 80,000,000 generations, Clade B ran for 10,000,000 generations. Bayesian skyline plots were constructed in TRACER, the data was exported and visualized using Excel. (PDF 144 kb)

Additional file 8: Bibliography of published invasion reports. (XLSX 30 kb)

## Abbreviations

*16S*: 16S ribosomal subunit
AF: Africa
AIC: Akaike information criterion
AMOVA: Analysis of molecular variance
ATL: Atlantic FWEC
BI: Bayesian inference
BSP: Bayesian skyline plot
C&S AM: Central and South America
COA: Coastal FWEC
COL: Colorado FWEC
*cox*1: Cytrochrome oxidase I gene
EU: Europe
FWEC: Freshwater ecological complex
GRB: Great Basin FWEC
H: Number of haplotypes
*Hd*: Haplotype diversity
IR: Invasive range
*ITS1*: Internal transcribed spacer region 1
K: Average sequence divergence
MCMC: Markov chain monte carlo
MD: Middle East
ME: Minimum evolution
MISS: Mississippi FWEC
ML: Maximum likelihood
MSB: Museum of Southwestern Biology
MSN: Minimum spanning network
mtDNA: Mitochondrial DNA
N: Number of individuals sampled
na: Not applicable
Ne: Effective population size
NR: Native range
NZ/OZ: New Zealand and Australia
pr: Prevalence
RIO: Rio Grande FWEC
S: Segregating site
S_O_: Observed species richness
STL: St. Lawrence FWEC
tsi: Time since invasion
USA: United States of America
Θw: Wattersons estimator per site
π: Nucleotide diversity
