# Supplementary figures and images for: Phylogeography and genetics of the globally invasive snail Physa acuta Draparnaud 1805, and its potential to serve as an intermediate host to larval digenetic trematodes
Source: BMC Evol Biol. 2018 Jul 3;18:103. doi: 10.1186/s12862-018-1208-z (PMC6029401; doi:10.1186/s12862-018-1208-z)

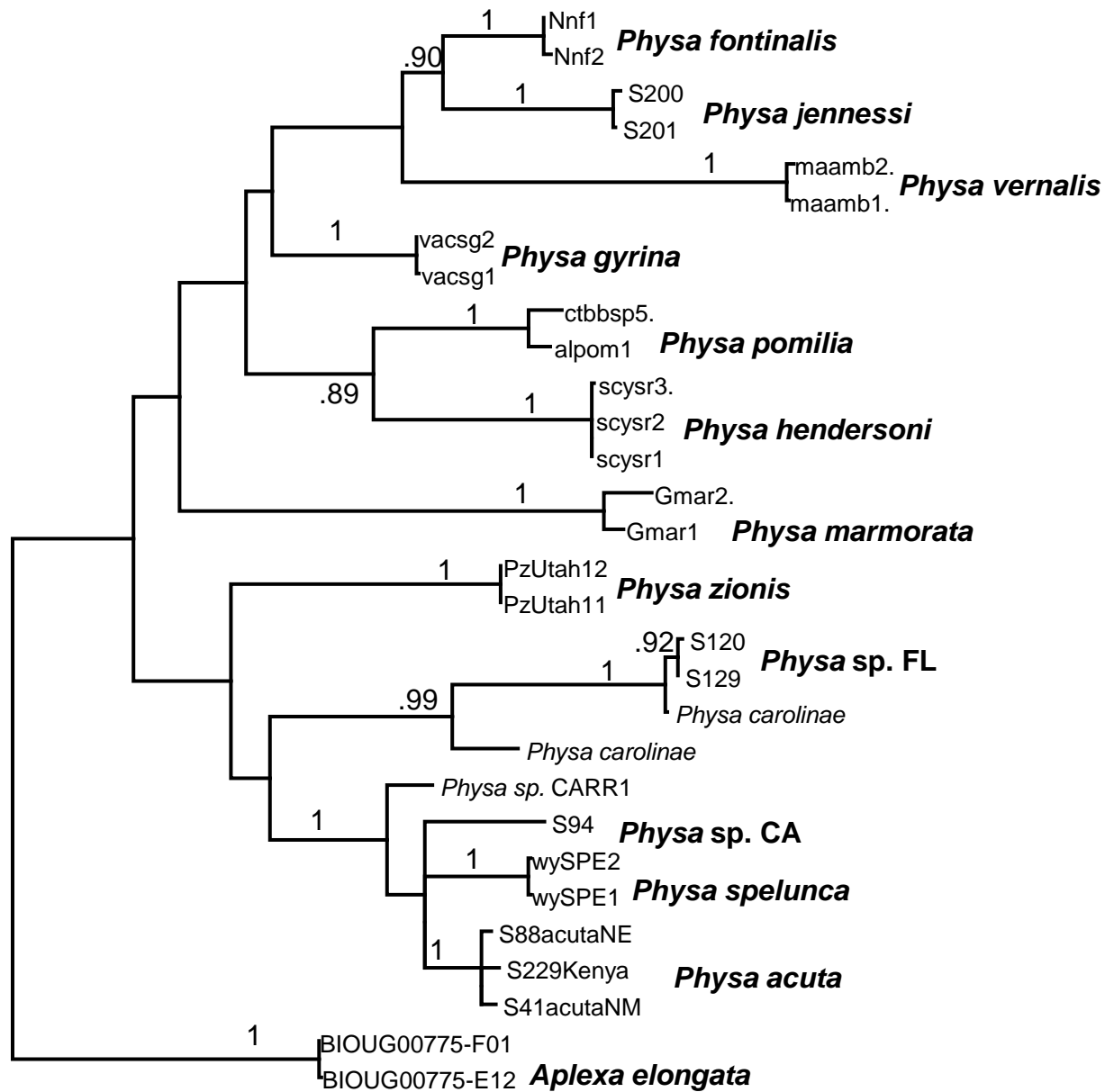

Supplement: Supplementary file 3 — CO1 phylogeny of Physa estimated using Bayesian inference. Posterior probability values ≥.95 are shown. Taxon names are reflective of the names assigned to sequences in the original NCBI records. (PDF 14 kb) [file 12862_2018_1208_MOESM3_ESM.pdf]

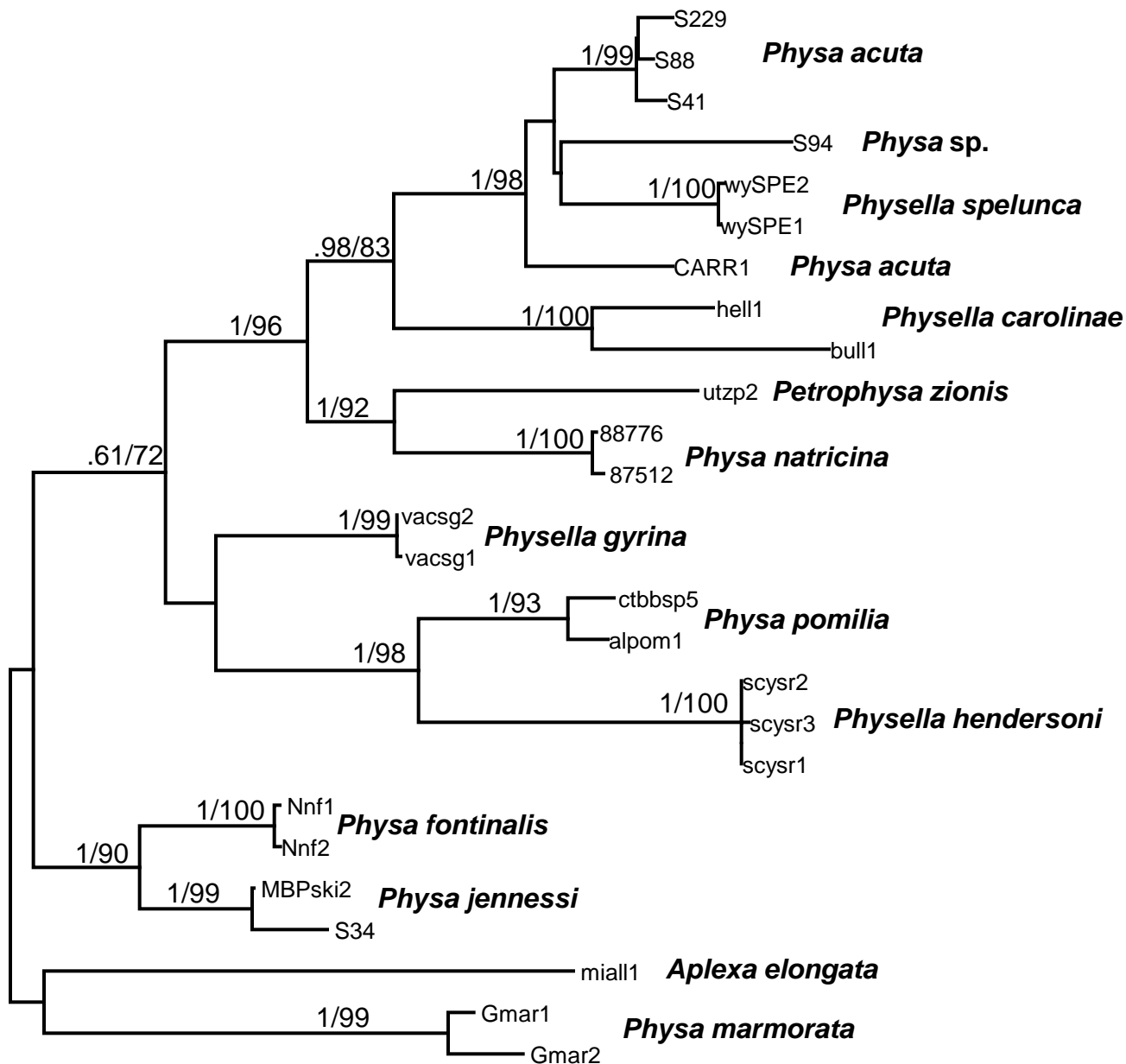

0.05

Supplement: Supplementary file 4 — Concatenated 16 + cox1 phylogeny. Branch support values ≥.95/70 (PP/BS) are shown. Taxon names are reflective of the names assigned to sequences in the original NCBI records. (PDF 165 kb) [file 12862_2018_1208_MOESM4_ESM.pdf]

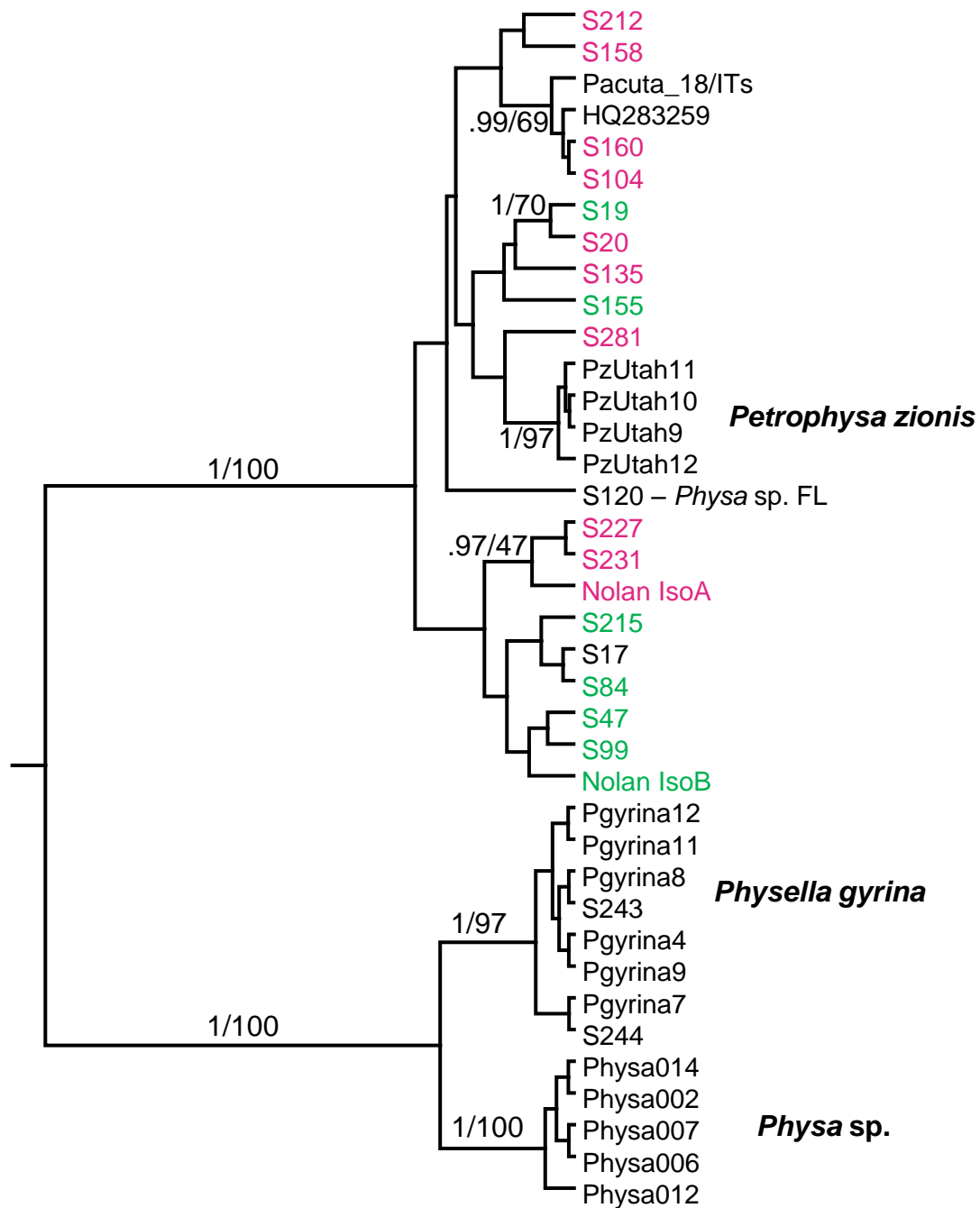

Supplement: Supplementary file 5 — ITS1 phylogeny estimated using Bayesian inference. Physa acuta samples are colored according to the clade they were recovered from based on mitochondrial gene tree analysis: pink = Clade A, green = Clade B. Physa acuta taxa labeled as black did not group within P. acuta based on mitochondrial gene trees, and were excluded from all in-group analyses (S17, S120 and all P. zionis samples). ITS1 data only exists for HQ283259 and Pacuta_18S/ITS and were therefore not included in mitochondrial gene tree analyses. Posterior probabilities are illustrated as branch support values. (PDF 143 kb) [file 12862_2018_1208_MOESM5_ESM.pdf]

- Rio Grande
- Coastal
- Colorado
- Mississippi
- Atlantic
- St. Lawrence
- Great Basin
- Invasive

A.

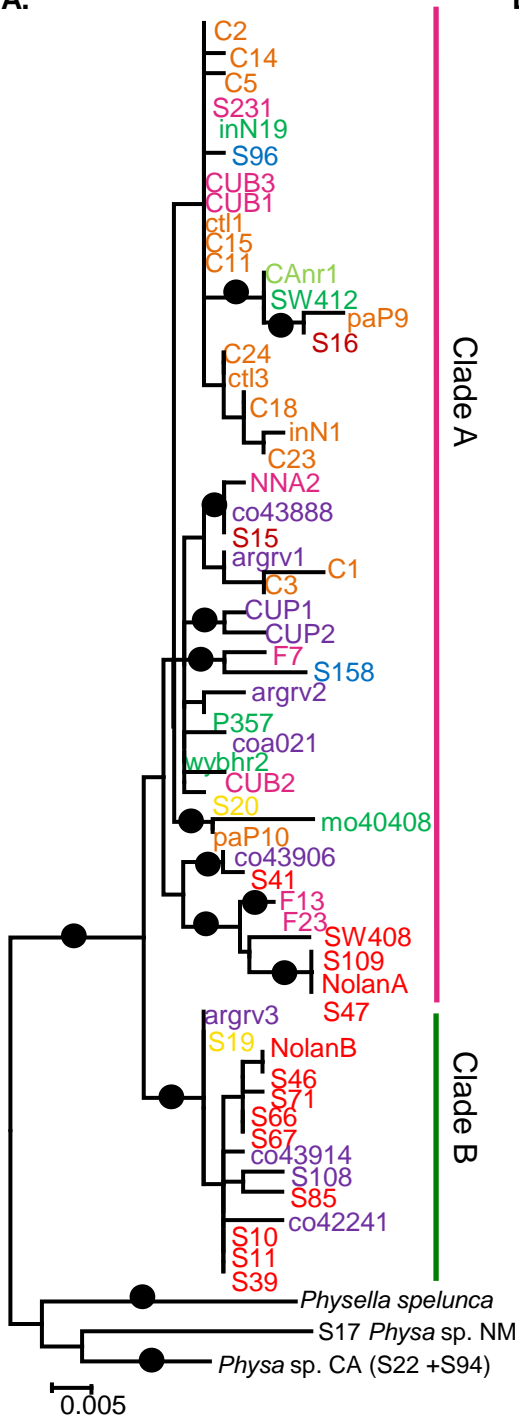

B.

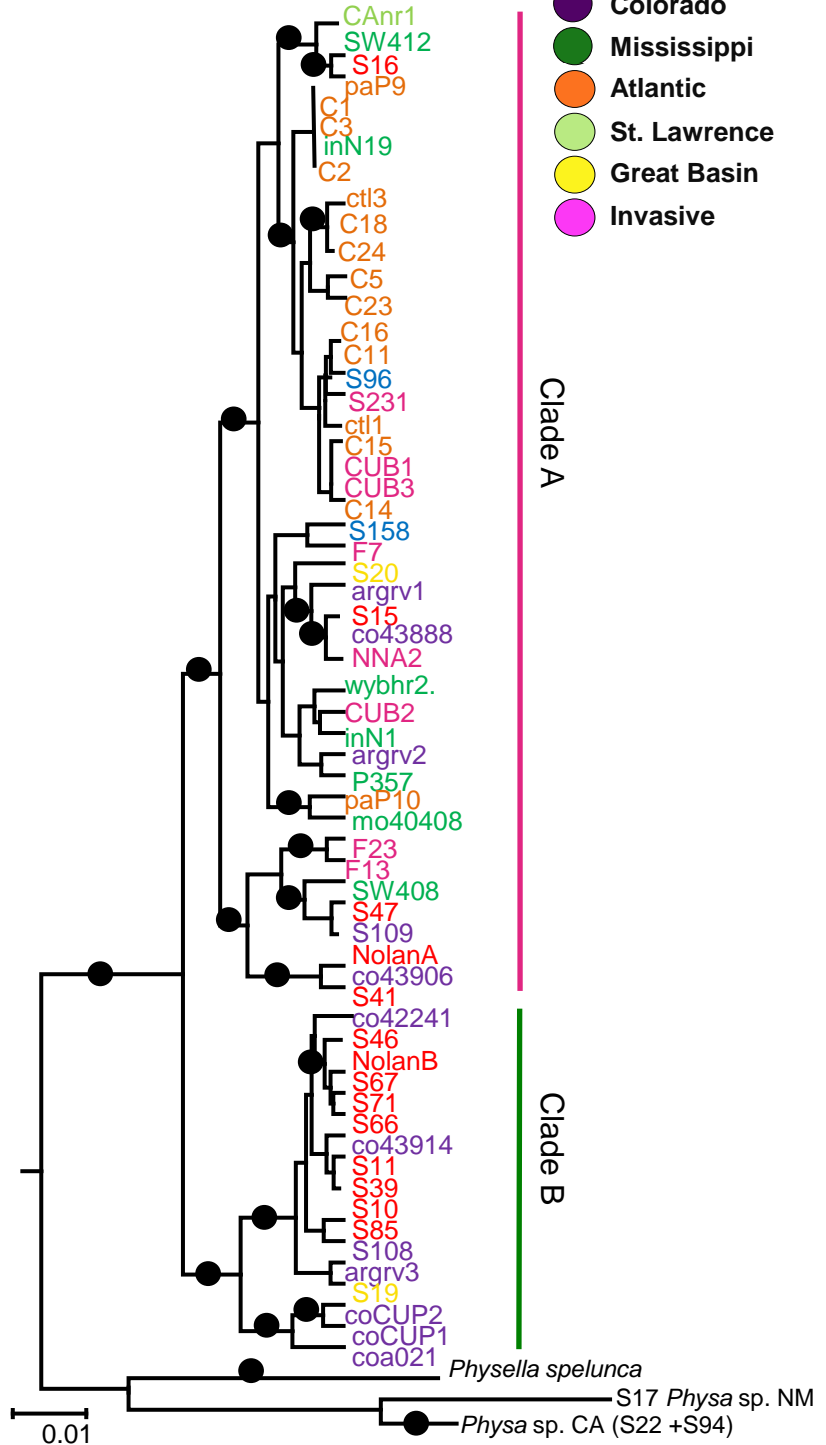

Supplement: Supplementary file 6 — 16S in-group analysis. Taxa are colored according to the FWEC they were collected from. Phylogeny (A) was generated using ML methods and phylogeny (B) was generated using BI. Branch support values ≥70 bootstrap (A) and ≥ .95 posterior probabilities (B) are denoted by a black circle. (PDF 188 kb) [file 12862_2018_1208_MOESM6_ESM.pdf]

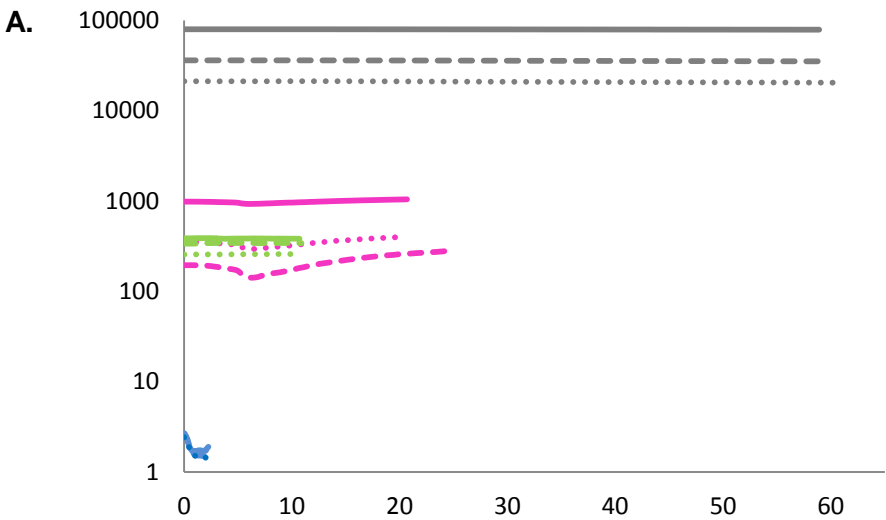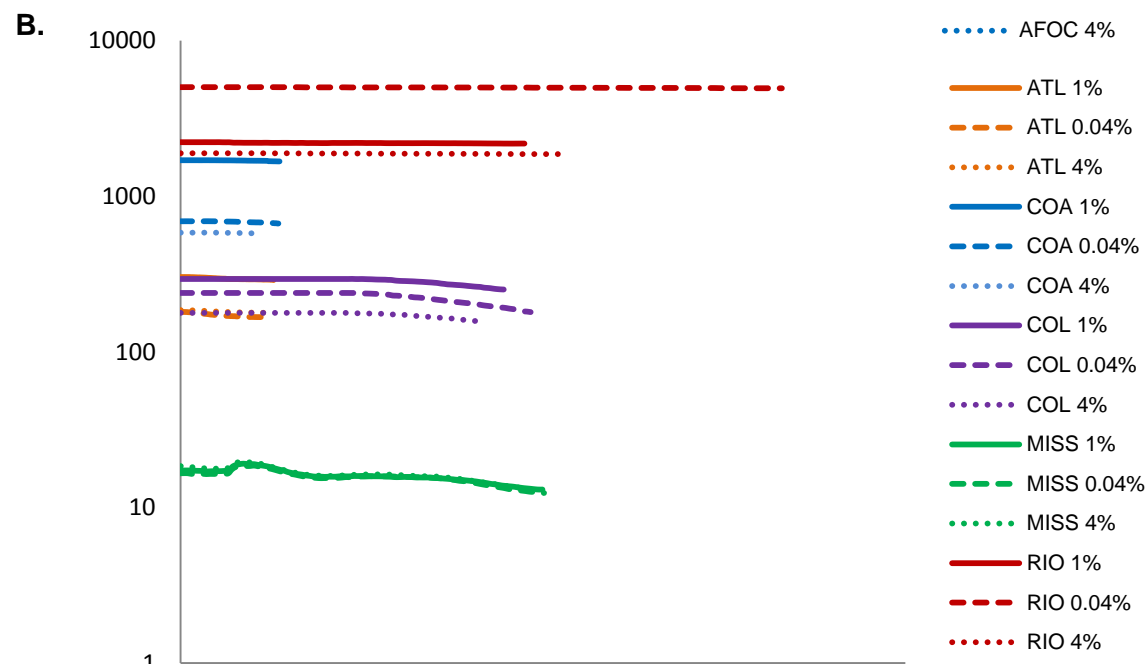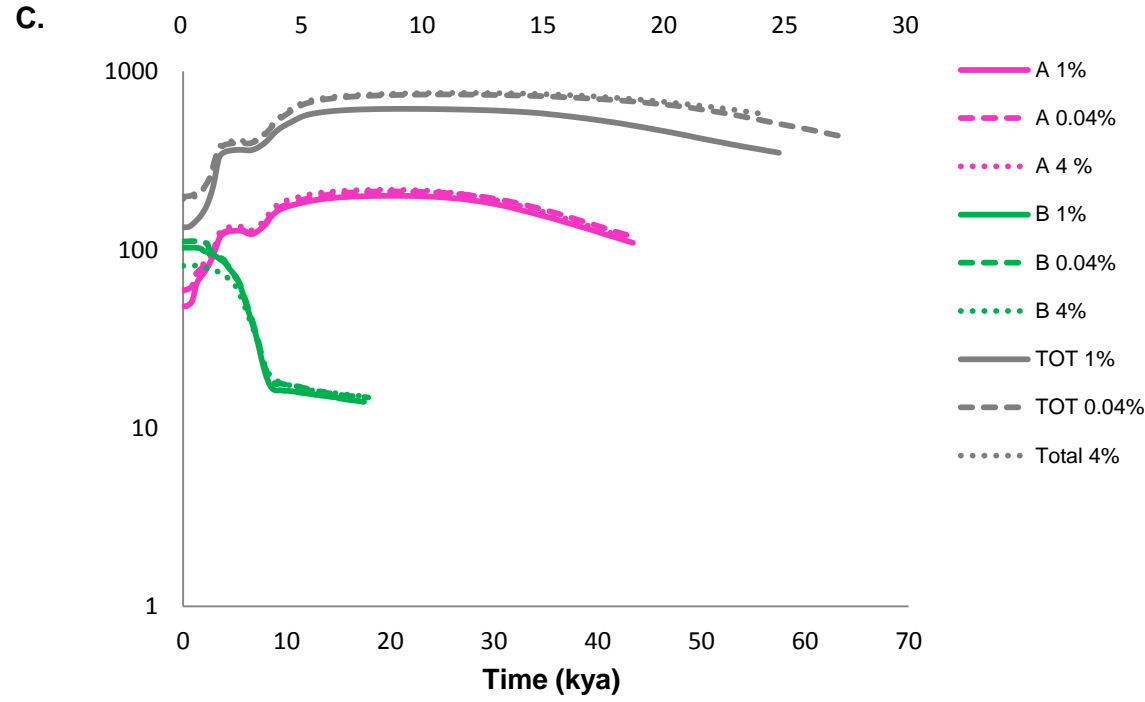

Supplement: Supplementary file 7 — Bayesian Skyline Plots. Effective size over time plots estimated in BEAST using a Bayesian Skyline prior, mean values are shown and confidence values were excluded for clarity. Three mutation rates (per million year): 1% (solid line), 0.04% (dashed line) and 4%(circle line) were tested. (A) Range-wide estimates for North America (NAM, dark grey, n = 79), Central and South America (SAM, lime green, n = 13), Eurasia (EUR, pink, n = 49) and Africa and Oceania (AFOC, blue, n = 7). North America dataset ran for 50,000,000 generations, Eurasia 20,000,000 generations, Central/South America and Africa/Oceania ran for 5,000,000 generations. B. Estimates by FWECs sampled: Rio Grande (RIO, red, n = 16), Coastal (COA, blue, n = 13), Colorado (COL, purple, n = 11), Atlantic (ATL, orange, n = 9), Mississippi (MISS, green, n = 26), the Great Basin and St. Lawrence FWECs were excluded because of low sample size. All FWEC datasets were run for 5,000,000 generations, with the exception of Atlantic (3000,000). C. Estimates of recovered Clades, Clade A (A, aqua, n = 116) and B (B, dark green, n = 33) compared to total (TOT, light grey, n = 149). The total dataset and Clade A ran for 80,000,000 generations, Clade B ran for 10,000,000 generations. Bayesian skyline plots were constructed in TRACER, the data was exported and visualized using Excel. (PDF 144 kb) [file 12862_2018_1208_MOESM7_ESM.pdf]
